# Bilinguals Use Language-Control Brain Areas More Than Monolinguals to Perform Non-Linguistic Switching Tasks

**DOI:** 10.1371/journal.pone.0073028

**Published:** 2013-09-13

**Authors:** Aina Rodríguez-Pujadas, Ana Sanjuán, Noelia Ventura-Campos, Patricia Román, Clara Martin, Francisco Barceló, Albert Costa, César Ávila

**Affiliations:** 1 Department of Psychology, Universitat Jaume I, Castelló de la Plana, Spain; 2 Department of Psychology, Penn State University, University Park, Pennsylvania, United States of America; 3 Department Technology, Universitat Pompeu Fabra, Barcelona, Spain; 4 Basque Center on Cognition, Brain and Language, Donostia, Spain; 5 IKERBASQUE, Bilbao, Spain; 6 Department of Psychology, University of the Balearic Islands, Palma, Balearic Islands, Spain; 7 Institució Catalana de Recerca i Estudis Avançats, Barcelona, Spain; University of Granada, Spain

## Abstract

We tested the hypothesis that early bilinguals use language-control brain areas more than monolinguals when performing non-linguistic executive control tasks. We do so by exploring the brain activity of early bilinguals and monolinguals in a task-switching paradigm using an embedded critical trial design. Crucially, the task was designed such that the behavioural performance of the two groups was comparable, allowing then to have a safer comparison between the corresponding brain activity in the two groups. Despite the lack of behavioural differences between both groups, early bilinguals used language-control areas – such as left caudate, and left inferior and middle frontal gyri – more than monolinguals, when performing the switching task. Results offer direct support for the notion that, early bilingualism exerts an effect in the neural circuitry responsible for executive control. This effect partially involves the recruitment of brain areas involved in language control when performing domain-general executive control tasks, highlighting the cross-talk between these two domains.

## Introduction

It is now well-accepted that the continuous and extensive training of language-control abilities in bilingual speakers affects the development and functioning of executive control (EC) systems. For example, bilinguals tend to outperform monolinguals in tasks that involve conflict resolution and monitoring, set shifting, etc. [1], [2], [3]. At present, however, much less is known about the impact of bilingualism on the brain organization of EC functions. Indeed, the few studies that have addressed this issue suggest that bilingualism does not only affect the efficiency of the EC functioning, but also the brain structures recruited when performing EC tasks [4], [5], [6], [7]. The main aim of the present study is to further advance in our knowledge of the impact of bilingualism on the brain networks involved in EC. In particular, we put to test the hypothesis that bilinguals will recruit language related areas when performing EC tasks to a larger extent than monolinguals.

It is widely accepted that the two languages of a bilingual are constantly active both when comprehending and producing language. Despite this co-activation, language control failures that lead to cross-language intrusions are scarce, at least in high-proficient bilinguals, revealing the excellent language control abilities develop by bilinguals. This language control system is sustained by a set of left lateralized brain areas [8]. Concretely, a brain network that involves the left inferior frontal gyrus, the left caudate, the left inferior parietal lobe and the anterior cingulate has been proposed as the main areas involved in language control. Therefore, as a result of the acquisition of two languages during early age, bilingual speakers continuously and extensively train this language control network.

Language control may be considered a special case of executive control. The mechanisms of executive control recruited to resolve competition between linguistic representations may be similar to those recruited when resolving competition between representations in perception and attention [9]. In fact, brain areas related to the control of executive processes are the bilateral inferior and medial frontal cortex, the caudate and the anterior cingulate [10], [11], and include those related to language control. In this context, it seems reasonable to hypothesise a cross-talk between the processes engaged in domain general executive control and those involved in language control. Indeed, such cross-talk is at the basis of current explanations of the observed bilingual advantage in executive control tasks that minimally involve language such as switching tasks and conflict resolution tasks [12]. A recent review of this behavioral evidence has served to outline the specific differences between monolinguals and bilinguals in executive processes [3]. They concluded that both groups did not differ in inhibitory processes, but bilinguals showed domain-general executive functioning advantages manifested in a more efficient processing when performed interference task. Importantly, these advantages are observed when the monitoring or attentional resources required to perform the task are high [2], [13].

Beyond the presence of such a cross-talk, our knowledge about the specific way in which bilingual language control alters the brain networks of individuals when performing non-linguistic executive control tasks is rather limited. One appealing possibility is that bilingual language control may impact in a qualitative (and not just in a quantitative) way the organization of cognitive control network, leading to the involvement of language-control brain areas in non-linguistic switching tasks [5]. In the present study, we intended to further test this original hypothesis by comparing the neural substrates of task switching in bilinguals and monolinguals in the absence of any group differences in task performance. This last feature is important, since any difference in the neural activation between these two groups could not be attributed to differences in behavioral performance, hence allowing a cleaner attribution to such brain modulation to the bilingual status of the participant.

With this purpose in mind, participants were submitted to a non-linguistic task-switching paradigm with low-monitoring demands [14]. Manipulation of the task's monitoring demands is relevant here because it has already been shown that the behavioural impact of bilingualism on EC tasks is reduced (or even absent) when the task at hand does not involve a high magnitude of the conflict effect [13]. However, given the early experience of bilinguals in managing two languages, we expected to find brain differences between groups even in absence of behavioral differences. In the sense, we hypothesize that language-control areas (i.e., left inferior frontal gyrus, left inferior parietal, left caudate and left ACC [15]) would be more activated in bilinguals than in monolinguals in the low-monitoring task switching of the present study. Comparing the two groups in a task that does not lead to differences in performance, as this will ease the interpretation of any potential brain differences between groups.

## Materials and Methods

### 1. Participants

The study was approved by Universitat Jaume I's ethics committee. Also, in accordance with the Universitat Jaume I's ethical protocol, each participant handed in a written informed consent to participating in the experiment and received a monetary reward for his participation.Thirty-six healthy right-handed undergraduates, including 18 early and high proficient Catalan-Spanish bilinguals (11 females, mean age  = 23.06, standard deviation (SD)  = 3.04) and 18 Spanish monolinguals (9 females, mean age  = 23.67, SD  = 4.28) gave written informed consent to participate in the study. There were no statistically significant between-group differences in age and gender. All participants had normal or corrected-to-normal vision, and were screened by self-report to exclude any subjects reporting previous or current neurological or psychiatric conditions, and current psychotropic medication use.

All participants were subjected to a preliminary interview about their use of languages, and their personal and familiar language history, after which they were assigned to the bilingual or monolingual groups. Age of acquisition was derived from a self-report questionnaire that contained questions about the frequency of use of each language at various ages from early life (1 =  only Spanish, 7 =  only Catalan). This questionnaire was structured into four main categories and three sub-categories: before primary school, primary school age (at school, home, free time), secondary school age (at school, home, free time), adult age (at work/university, home, free time), and the corresponding questions intended to assess the extent of early and continuous practice in Catalan and Spanish. All the bilingual participants had learned both languages and had sufficient experience with them for the first four years of life: 13 bilinguals learned both languages at home and at school, whereas the 5 remaining bilinguals learned Spanish at home and Catalan at school. All bilinguals reported a continuous use of both languages since they were 4 years old. Despite this early and continuous exposure, some participants showed preferences for one of their languages. We took their preferences to establish the participant's L1 (or dominant) and L2 (or non-dominant) (10 participants preferred Catalan, 3 Spanish, and 5 had no preference for either language).

Participants self-rated their language proficiency on a 4-point scale (1 =  “very low proficiency,” 4 =  “very proficient”) in four different domains: listening, reading, speaking and writing. Given the very extensive and early experience, all bilingual participants rated 4 (“very proficient”) in all these domains in both Spanish and Catalan. It is important to note that these participants received bilingual schooling for at least 13 years. This bilingual schooling does not mean that Catalan or Spanish was taught as a foreign language. Instead, different courses of the syllabus (e.g., maths, social sciences, etc.) were taught in different languages. Thus, all bilinguals had a perfect command of both languages at all levels and self-reported the need for making continuous changes between Spanish and Catalan depending on the interlocutor present. Noteworthy, subjective measures of self-reported language proficiency appear to provide an effective measure of bilingual ability [16] (Marian et al. 2007). This is probably even more so for very homogeneous samples like the one tested in this study. Bilinguals also self-reported an actual active use of both languages throughout their life (mean 59% use of Catalan and 41% use of Spanish), and that they frequently switch between languages depending on environment rules.

The monolingual group was formed by Spanish participants coming from monolingual regions of Spain who had moved to Castelló or Valencia less than a year before the experiment started and who stated that they had a very low proficiency in Catalan. It is important to remind that in the Comunidad Valenciana Catalan is used by 30% of the population, while Spanish is used by all the population. Moreover, although all the bilingual and monolingual participants had studied English as a second (or third) language at school, none of them showed good proficiency in English according to self-reported questionnaires.

### 2. Task

A non-linguistic switching task devised by Barceló [14] was adapted to the fMRI scanner using an embedded critical trial design [17] based on an intermittently-instructed task-cueing paradigm (switching task). Visual stimuli consisted in four equiprobable coloured shapes (red and blue circles and squares; p = 0.225 each) that were embedded within two infrequent black shapes that were the events of interest (vertical dollar symbol and horizontal dollar symbol; p = 0.05 each). The inter-stimulus interval (ISI) was set at 1500 ms and each stimulus lasted 500 ms. Each event of interest (i.e. a dollar symbol) was separated by a varying number of coloured shapes (from 6 to 9) with a temporal mean duration of 14.5 seconds (SD  = 4.04) to adequately observe the hemodynamic response for each event and to ensure that event-related responses did not overlap.

Before the scan session, subjects completed a 5-minute practice session to ensure they understood the instructions. In the experimental session, each subject performed a total of 70 trial sequences distributed into five runs (each run consisted of 14 sequences, 98 volume acquisitions and lasting 4∶05 minutes). The speed and accuracy of each behavioural response were registered.

**Figure 1:**
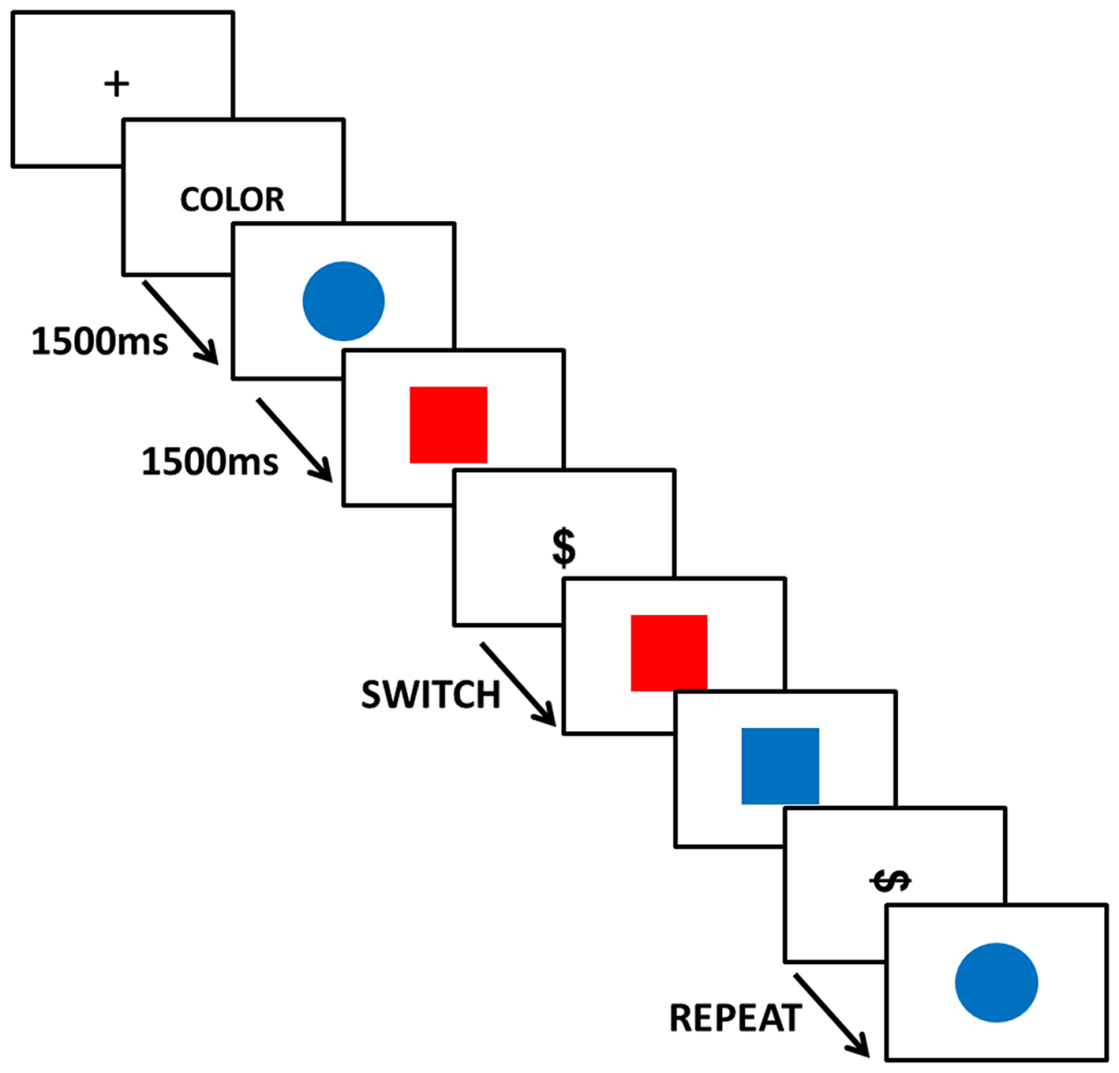
Task. Schematic of the serial visual presentation of stimulus displayed during the scan session.

The task consisted in classifying four coloured shapes (red and blue circles and squares; 185 of each kind of shape in the five runs) according to two classification rules (either colour or shape) by pressing a response button with their right index and thumb (370 responses with each finger in the five runs). When sorting by the colour rule, subjects pressed the index button for “red” and the thumb button for “blue”. When sorting by the shape rule, subjects pressed the index button for “circle” and the thumb button for “square”. At the beginning of a run, a written cue was presented to indicate the initial response rule (“COLOR” for colour and “FORMA” for shape). Next, two black symbols (dollar symbol horizontally or vertically) instructed subjects to either “switch” or “repeat” the previous response rule, respectively. So, there were two events of interest: switch and repeat (See Figure 1).

In order to avoid an inaccurate scoring of task-switching errors at rule transition points (after a black symbol), the first and last shapes in a trial sequence always consisted in either a red square or a blue circle. This allowed for an unambiguous assignment of motor responses with the correct or incorrect classification rules. Besides, the manual response was counterbalanced (the same number of manual responses were done with both fingers) for the first and the second response after a black figure, and the same was done for the previous and the following response also in the case that the participant used the incorrect response rule. Moreover, in order to keep perceptual priming effects constant across conditions, the sequential probabilities between each pair of stimuli were controlled. The global probability of two successive repeat cues was the same as that of two successive switch cues. Likewise, the global probability of alternations between switch-repeat cues equalled the probability of repeat-switch alternations. Finally, both black symbols instructed the same number of switch and repeat trials (35 of each in the five runs).

The stimulus material in this task was chosen to keep working memory demands and novelty effects at a minimum. In particular, the six perceptual items employed in the task could be easily memorized and discriminated from each other, and they were selected to meet the theoretical limits of human capacity for selecting and holding information in working memory [18]. The task was programmed and presented using the Presentation software (Neurobehavioral System, Inc., Albany, CA), implemented in Microsoft XP operating system. Visual stimuli were displayed inside the scanner using Visuastim goggles (Resonance Technologies, Inc.), their presentation was synchronized with a scan through a SyncBox (Nordic NeuroLab AS, Bergen, Norway), and responses were registered with a recording device ResponseGrip (Nordic Neurolab AS, Bergen, Norway).

### 3. Behavioural measures

The percentage of errors was calculated for each task condition and reaction times (RTs) were obtained from correct trials only. The switch cost was computed as the difference in the mean RTs between the first targets following switch and repeat cues [19], [14]. Mean RTs and errors were subjected to repeated measures analyses of variance (ANOVAs) with Trial Type (repeat vs. switch) as the within-subject factor and Group (bilinguals vs. monolinguals) as the between-subjects factor. Only the first target after the cue was included in the analyses as first target trials present maximal effects of switch-specific local costs [19], [14].

### 4. FMRI parameters

All the experimental sessions were performed in a 1.5T scanner (Siemens Avanto, Erlangen, Germany). Participants were placed inside the scanner in the supine position. A sequence BOLD echo planar imaging (BOLD-EPI) of 98 volumes per run was used for fMRI (TE  = 50 ms, TR  = 2500 ms, FOV  = 224×224, matrix  = 64×64, voxel size  = 3.5×3.5×4, 3.5-mm slice thickness, gap  = 0.5-mm, Flip  = 90°). We acquired 28 interleaved axial slices parallels to the anterior-posterior commissure (AC-PC) plane covering the entire brain. Prior to the functional MR sequence, an anatomical 3D volume was acquired by using a T1-weighted gradient echo pulse sequence (TE  = 4.9 ms, TR  = 11 ms, FOV  = 24 cm, matrix  = 256×224×166, voxel size 1×1×1, 1-mm slice thickness).

### 5. Image analyses

Image processing and statistical analyses were carried out using SPM5 (Wellcome Trust Centre for Neuroimaging, London, UK). Each participant's scans were first temporally aligned across the brain volume by slice-timing correction, and then the images were realigned and resliced to the mean image for head motion. Then they were coregistered with the corresponding anatomical (T1-weighted) image, and were normalized (voxels rescaled to 3 mm^3^) with the normalization parameters obtained after anatomical segmentation within a standard stereotactic space (the T1-weighted template from the Montreal Neurological Institute, MNI) to present functional images in the coordinates of a standard stereotactic space. Finally, functional volumes were smoothed using an 8-mm FWHM Gaussian kernel. Image analyses were performed by means of a General Linear Model approach. In the first-level analysis, the five runs were analyzed for each subject by modelling switch events and repeat events separately after convolving each event-related unit impulse with a canonical haemodynamic response function and its first temporal derivative. Realignment parameters were included for each subject as regressors of non interest. A high-pass filter (128 s) was applied to the functional data to eliminate low-frequency components. From this first level, we computed con-images of the parameter estimates to make a comparison between the switch and repeat conditions (switch > repeat) at each voxel for each subject. The resulting images of the parameter estimates were used in the second-level random effect analysis to explore the average effects within groups and the differences between them. At this second level, we performed one sample t-test for each linguistic group (bilinguals and monolinguals). In this analysis, correction for multiple comparisons was done at the cluster level [20]. The results were reported at a *p*<0.05 FWE correction at the cluster level (a voxel-level threshold of *p*<0.001). A two-sample t-test was done to compare between the two linguistic groups (bilinguals and monolinguals). For this analysis, specific regions of interest (ROIs) associated with both cognitive and language control, were studied (see [21]): the right and left inferior frontal gyrus, the right and left caudate and the ACC. These ROIs (spheres of 5 mm radius) were centered on the areas identified in a one sample t-test performed for all the participants (see Table S1 and Figure S1). The statistical threshold was set at *p*<0.05 FWE corrected.

## Results

### 1. Behavioural performance

Mean RTs (bilinguals: 387 ms (SD  = 49); monolinguals: 420 ms (SD  = 70)) and error rates (bilinguals: 7.6% (SD  = 2.9); monolinguals: 10.2% (SD  = 6.1)) were not significantly different between the two groups of participants.

**Figure 2:**
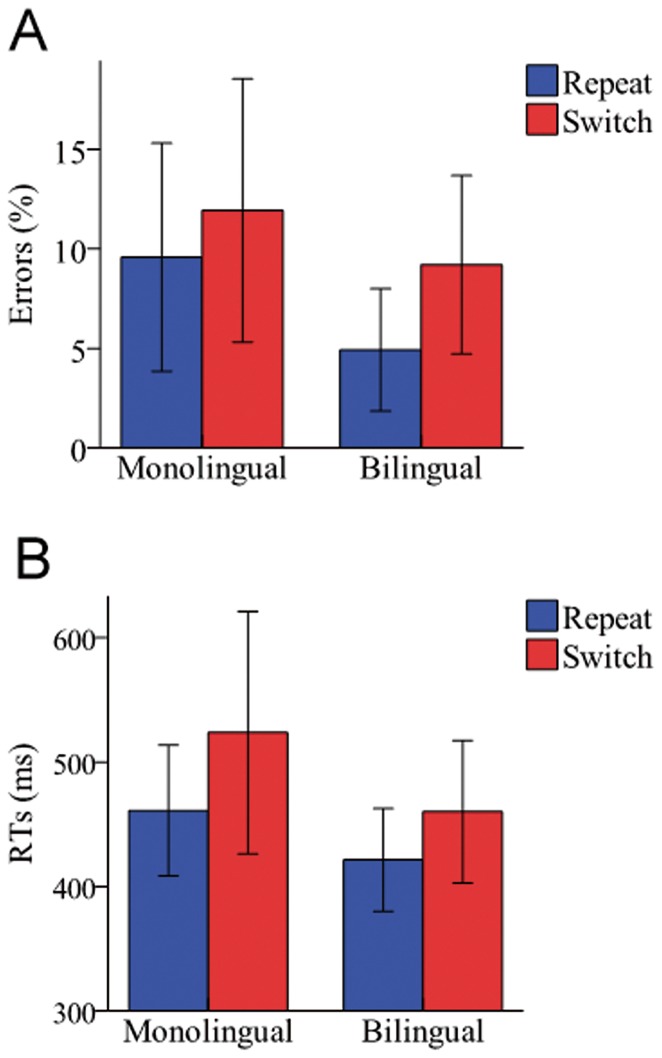
Magnitude of switch costs in errors (A) and RTs (B). Magnitude of switch costs for bilinguals and monolinguals in a percentage of errors (A) and mean RTs in milliseconds (B). Switch costs were analysed by comparing the responses in the first target trial following repeat and switch cues. As expected, the ANOVAs did not reveal any significant difference between bilinguals and monolinguals (*p*>0.10).

Switch costs were analysed by comparing the responses in the first target trial following repeat and switch cues. The main trial effect was significant in both errors (*F* (1, 34)  = 9.37, *p*<0.05) and RTs (*F* (1, 34)  = 16.92, *p*<0.001). That is, the target responses following a switch cue elicited longer RTs and higher error rates than the target responses following a repeat cue. It is important to note, however, that the interaction between Trial and Group variables was not significant; (*F* (1, 34)  = 0.23, *p*>0.10 for errors; and *F* (1, 34)  = .91, *p*>0.10 for RTs); indicating that the magnitude of the switch cost was similar for both groups of participants (see Figure 2).

### 2. Imaging data

#### 2.1 Within-group activations

**Figure 3:**
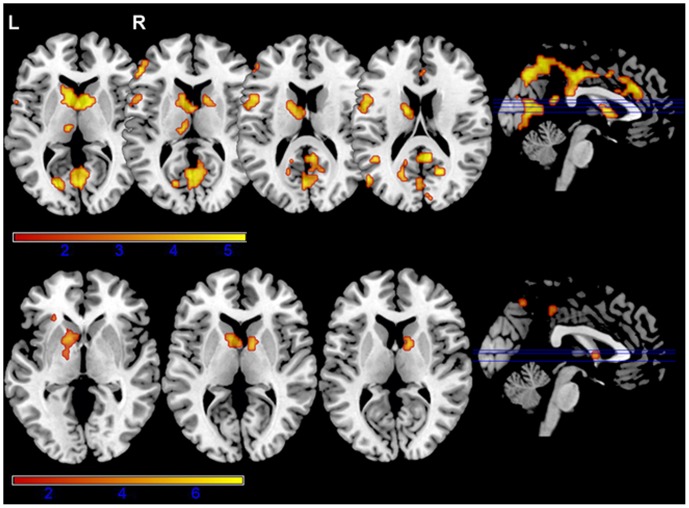
Brain activations for each group in the comparison between switch and repeat trials. Brain activations for bilinguals (top) and monolinguals (bottom) in the comparison between the switch and repeat trials (one-sample t-test at *p*<0.05, FWE cluster-corrected). Results for both groups showed the participation of both caudates, and the inferior frontal gyrus.

In these analyses, we assessed the differences in brain activation between the switch and repeat trials as an index of switch costs for each group (see Table 1 and Figure 3). In the bilingual group, the comparison made between the switch and repeat trials showed a cluster of activation with a peak in correspondence of the head of the left and right caudates, the left and right cingulate gyri and the left inferior and middle frontal gyri (*p*<0.05, FWE cluster-corrected). In the monolingual group, the same comparison (switch vs. repeat trials) showed that the areas mainly activated were in the basal ganglia (bilateral caudate head and bilateral globus pallidus) and the left inferior frontal gyrus (*p*<0.05, FWE cluster-corrected).

**Table 1:** Brain activations for bilinguals and monolinguals in the comparison between the switch and repeat trials.

BILINGUALS						
Activated regions	Brodman Area	Talairach coordinates			T-value	Cluster Size(mm^3^)
		x	y	z		
**L, Superior Frontal Gyrus**	6	−21	11	52	6.91	8802
**L, Middle Frontal Gyrus**	6	−18	6	58	6.61	
**L, Middle Frontal Gyrus**	9	−27	42	34	6.11	2106
**L, Middle Frontal Gyrus**	9	−24	34	34	5.35	
**L, Middle Frontal Gyrus**	9	−33	42	26	5.29	
**L, Middle Frontal Gyrus**	46	−42	44	6	4.26	
**L, Medial Frontal Gyrus**	6	−12	−23	56	5.98	1998
**L, Medial Frontal Gyrus**	6	−6	−11	67	3.88	
**L, Inferior Frontal Gyrus**	46	−50	35	9	5.52	648
**L, Precentral Gyrus**	6	−42	−1	47	6.65	
**L, Precentral Gyrus**	6	−15	−17	62	5.92	
**L, Inferior Parietal Lobule**	40	−45	−48	25	7.82	945
**L, Middle Temporal Gyrus**	39	−42	−57	22	3.72	
**L, Middle Temporal Gyrus**	39	−50	−66	23	5.98	621
**L, Middle Temporal Gyrus**	39	−45	−74	26	4.66	
**L, Middle Temporal Gyrus**	21	−56	−50	−3	5.47	621
**L, Cingulate Gyrus**	32	−6	22	40	5.24	
**L, Caudate Head**		−6	12	5	6.84	
**R, Middle Frontal Gyrus**	6	18	6	61	6.28	783
**R, Precuneus**	7	18	−65	36	9.73	39960
**R, Precuneus**	31	18	−60	22	7.13	
**R, Precuneus**	7	36	−71	42	6.03	729
**R, Middle Occipital Gyrus**	19	48	−76	4	6.06	621
**R, Middle Occipital Gyrus**	19	48	−73	−4	4.17	
**R, Anterior Cingulate**	32	3	33	23	6.78	4995
**R, Cingulate Gyrus**	32	15	11	38	5.19	
**R, Posterior Cingulate**	30	3	−46	19	6.83	
**R, Caudate Head**		6	9	2	8.14	10962
**R, Putamen**		12	9	−3	7.29	

MONOLINGUALS						
**L, Inferior Frontal Gyrus**	47	−24	23	−9	4.49	
**L, Paracentral Lobe**	31	−3	−30	43	5.02	756
**L, Precuneus**	31	−27	−71	28	5.31	918
**L, Precuneus**	7	−9	−71	37	5.22	1215
**L, Precuneus**	7	−9	−59	53	4.43	
**L, Inferior Parietal Lobule**	40	−50	−33	40	4.90	999
**L, Temporal Lobe**	37	−48	−47	−5	5.37	1215
**L, Middle Occipital Gyrus**	19	−50	−56	−7	5.29	
**L, Middle Occipital Gyrus**	19	−27	−78	20	5.26	
**L, Caudate Head**		−6	9	2	7.29	3105
**L,Medial Globus Pallidus**		−15	−6	3	6.29	
**R,Precuneus**	7	12	−59	50	4.95	1350
**R,Lateral Globus Pallidus**		12	3	5	6.10	999
**R, Caudate Body**		15	1	17	3.87	

Note: One-sample t-test at *p*<0.05, FWE cluster-corrected.

#### 2.2Between-groups comparison

**Figure 4:**
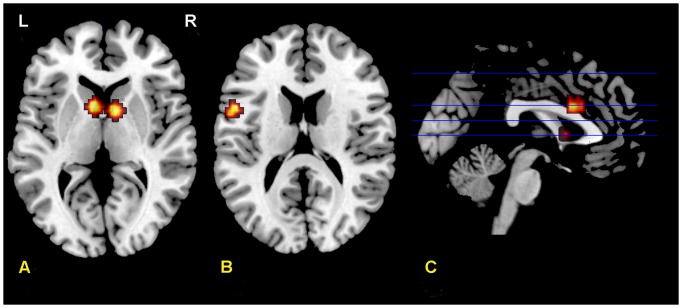
ROIs used for the two-sample t-test. Axial and sagittal sections showing the four ROIs used for the two-sample t-test analyses: left and right caudate (A), left inferior frontal gyrus (B) and the ACC (C). These ROIs (spheres of 5 mm radius) were centered on the areas identified in a one sample t-test performed for all the participants (see Table S1 and Figure S1). Results of the two-sample comparison of bilinguals and monolinguals appear on Table 2.

We selected from Table S1 the coordinates for ROIs (see Figure 4). The between-groups comparison using a two-sample t-test revealed that, compared to monolinguals, bilinguals showed increased brain activity in the left caudate (81 mm^3^), and the left inferior frontal gyrus (135 mm^3^) (see Table 2). However, differences were not significant for the ACC, the right inferior frontal gyrus and the right caudate. When we lowered the threshold (*p*>0.005 uncorrected), whole brain analysis showed similar results (see Table S2 and Figure S2). The inverse comparison (monolinguals vs. bilinguals) yielded no significant brain differences in the areas of interest.

**Table 2:** Region of interest showing increased activation for bilinguals compared with monolinguals.

Region of interest	Talairach coordinates			T-value	Significance
	x	y	z		
**L, Inferior Frontal Gyrus**	−54	6	21	3.17	*p*<0.05, FWE-corrected
**L, Anterior Cingulate Cortex**	3	18	24	1.15	ns
**L, Caudate**	−9	9	9	3.41	*p*<0.05, FWE-corrected
**R, Caudate**	6	9	0	1.62	ns

## Discussion

In the present study, early bilinguals and monolinguals performed a non-linguistic switching task with low-monitoring demands, which involved runs of target trials intermittently interrupted by the presentation of instructional cues. That is, the task cue was not presented in each trial. Arguably, this task involved less monitoring demands than the task-switching paradigms in which each target trial is preceded by a task cue.

Crucially, to minimize the involvement of verbal components, non verbal symbols as cue and manual responses were used; whereas that, main verbal components of the task use the same word in Spanish and Catalan (Shape, Color, switch, repeat...). Importantly, as the contrast of interest compared Switch and Repeat trials, the possible role of language control, internal verbalizations and L2 inhibition were also controlled.

Participants' performance in this task showed a significant switch cost in terms of RTs and error rates. It is noteworthy, however, that the magnitude of the switch cost was comparable in bilinguals and monolinguals. That is, with this task-switching paradigm, in which monitoring demands are low, the magnitude of the behavioural switch cost did not seem to be affected by bilingualism. However, we observed differences between the groups in brain activities associated with the switch cost, which is precisely the crucial contribution of the present study. Bilinguals displayed greater activation than monolinguals specifically associated with task switching in the left caudate and in the left inferior frontal gyrus. Therefore, the present results are consistent with our previous observations [5] in which early bilinguals used brain areas involved in language control to a larger extent than monolinguals during non-lingusitic task switching. The important point here is that this observation was still present even in the absence of statistically significant behavioural differences between the two groups. Thus, neural differences observed in the previous [5] and in the present study suggest that bilinguals use language areas more than monolinguals to perform switching tasks. Whether to not these brain differences between bilinguals and monolinguals leads to behavioural differences in task switching appears to depend on various properties of the specific task-switching implementation. For example, when the task switch requires high cognitive demands and the need of endogenous rapid disengaging from irrelevant inputs ([2], [13], [3], [22]) then it seems to be more likely to find these behavioral differences.

Bilinguals and monolinguals display greater activity in the caudate nucleus bilaterally when processing switch vs. repeat trials. This activity is in accordance with the notion that the caudate nucleus is the main structure involved in shifting between already established task sets, especially when the monitoring and inhibitory demands of the task are low [10]. Indeed, damage to basal ganglia structures leads to difficulties in task-switching paradigms, as shown in Huntington's disease [23] and Parkinson's disease [24]. Yet, on top of this bilateral activity associated with task-switching in both groups, we also observed a difference between the groups. Although both monolinguals and bilinguals recruit the left caudate when switching, bilinguals do so to a greater extent than monolinguals. The origin of such a bilingual effect probably lies in the involvement of the left caudate nucleus in bilingual language control. In other words, although the left caudate appears to be involved in monolingual language processing [25], its involvement in bilingual language control is even more fundamental [26], [27], [28], [29], [30].For example, damage to this area leads to pathological language switching [31], [32]. Hence given the specific involvement of this area in bilingualism, it is reasonable to expect functional differences also in task switching in accordance with the participants' bilingual status, and this is precisely what we found. Consequently, a likely explanation for this greater left caudate involvement in bilinguals could be its extension from the linguistic function underlying language switching to also encompass the attentional control required to select the correct response set in a non-linguistic task-switching paradigm.

Further evidence for linguistically related areas being more recruited by bilinguals than monolinguals during task switching originates from the pattern of activity observed in the left inferior frontal gyri. This area is activated to a greater extent in switch than in repeat trials both in bilinguals and monolinguals. This observation is congruent with previous neuropsychological studies reporting that damage to this area leads to reduced endogenous control in task switching which, in turn, leads to exaggerated sensitivity to the exogenous cueing of the task set [10]. Confirming our previous result [5], we found that the left inferior frontal gyrus is more activated in bilinguals than in monolinguals. As for the left caudate, this area is involved in language processing in both monolinguals and bilinguals, and it also plays a key role in bilingual language control [21]. Hence, the fact that bilinguals recruit the left inferior frontal gyrus more than monolinguals suggests that continuous bilingual language control has an effect on the extent to which linguistically dedicated areas are involved in task switching.

There are, however, certain limitations in our study that should be addressed in further studies. First, our sample is formed by early and highly proficient of bilinguals that live in a society with frequents demands of language switching. Future research should determine if our conclusions can be generalized to other kind of bilinguals with different characteristics. Second, the monolingual sample used in this study was formed by undergraduates coming from other parts of Spain. The lack of control of sociodemographic variables should not be discarded a possible bias of the results [33], although previous studies offer strong support for the claim that bilingualism acts independently of variables such as language similarity, cultural background, and language of schooling in influencing nonverbal outcomes [34], [35]. Third, even although the task could be carried out without using linguistic representations, we cannot completely rule out that participants engaged in some sort of implicit linguistic behaviour. If so, then brain differences between monolinguals and bilinguals in this experiment may be driven by a different implicit use of these strategies.

Overall, these results suggest that bilinguals and monolinguals recruit largely similar brain areas to perform non-linguistic task-switching. However, there are relevant differences associated with bilingualism. It appears that bilinguals engage to a greater extent than monolinguals the brain areas associated with language control, in switching tasks where language is not involved. This suggests that there is some cross-talk between the brain areas housing language control and those involved in the general-purpose cognitive control system [36]. This is likely to happen as a result of bilinguals' very early experience in managing and controlling two languages. Furthermore, the fact that these brain differences are observed even in the absence of behavioural differences between the two groups suggests that the functional brain dynamics associated with bilingualism do not necessarily lead to more efficient behavioural performance. In other words, we herein describe how bilinguals and monolinguals attain comparable performance levels in a low-monitoring demand task, even though they recruit the same brain areas to a different extent. In fact, we may even argue that the brain control exerted by monolinguals in the present task is even more efficient than for bilinguals. Future studies should determine if this functional reorganization depends on factors such as age of acquisition or the amount of previous experience in language switching [29].

## Supporting Information

Figure S1 **Brain activations for all participants in the comparison between switch and repeat trials (one-sample t-test at** ***p*** **<0.05, FWE cluster-corrected).**
(TIF)

Figure S2 **Brain activations for bilinguals compared with monolinguals in the comparison between switch and repeat trials (two-sample t-test at** ***p*** **<0.005 uncorrected).**
(TIF)

Table S1 **Brain activations for all participants when comparing between switch and repeat trials.**
(DOCX)

Table S2 **Region of interest showing increased activation for bilinguals compared with monolinguals.**
(DOCX)
