# Supplementary figures and images for: Bilinguals Use Language-Control Brain Areas More Than Monolinguals to Perform Non-Linguistic Switching Tasks
Source: PLoS One. 2013 Sep 13;8(9):e73028. doi: 10.1371/journal.pone.0073028 (PMC3772880; doi:10.1371/journal.pone.0073028)

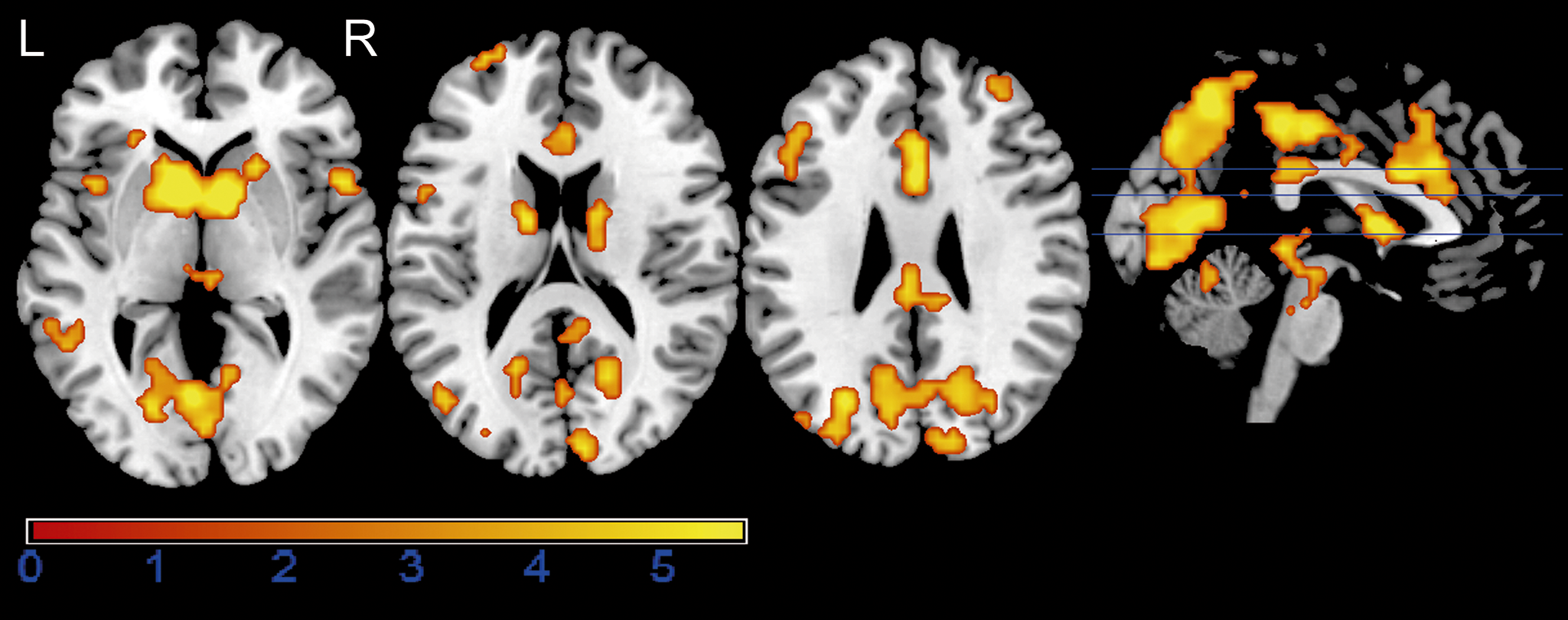

Supplement: Figure S1 — Brain activations for all participants in the comparison between switch and repeat trials (one-sample t-test at p <0.05, FWE cluster-corrected). (TIF) [file pone.0073028.s001.tif]

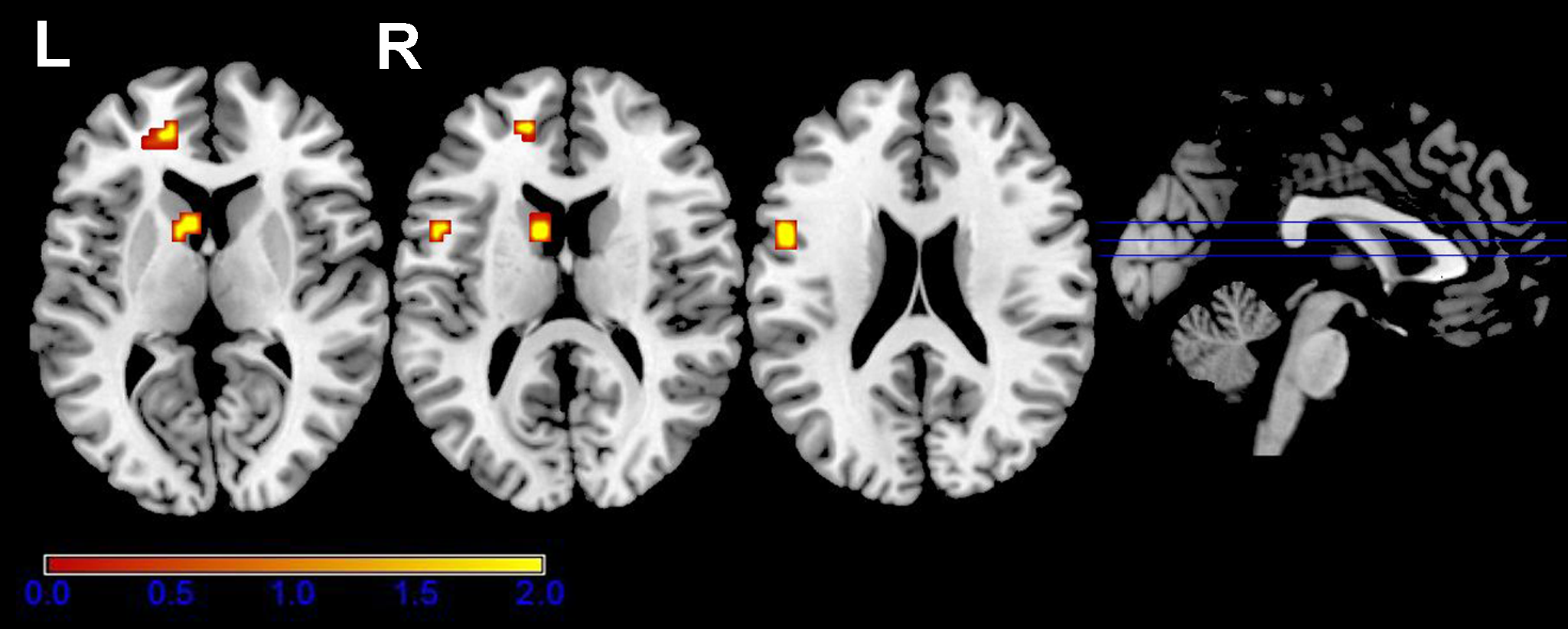

Supplement: Figure S2 — Brain activations for bilinguals compared with monolinguals in the comparison between switch and repeat trials (two-sample t-test at p <0.005 uncorrected). (TIF) [file pone.0073028.s002.tif]
