# Supplementary material for: Bilinguals Use Language-Control Brain Areas More Than Monolinguals to Perform Non-Linguistic Switching Tasks
Source: PLoS One. 2013 Sep 13;8(9):e73028. doi: 10.1371/journal.pone.0073028 (PMC3772880; doi:10.1371/journal.pone.0073028)
Supplement: Table S1 — Brain activations for all participants when comparing between switch and repeat trials. (DOCX) [file pone.0073028.s003.docx]

| **Activated regions** | **Brodmann Area** | **Talairach coordinates** | | | **T-value** | **Cluster Size (mm³)** |
| --- | --- | --- | --- | --- | --- | --- |
|  |  | **x** | **y** | **z** |  |  |
| **L, Superior Frontal Gyrus** | 10 | -30 | 59 | 14 | 5.07 | 729 |
| **L, Medial Frontal Gyrus** | 6 | -21 | 8 | 49 | 6.29 | 5373 |
| **L, Middle Frontal Gyrus** | 9 | -50 | 16 | 32 | 5.63 |  |
| **L, Middle Frontal Gyrus** | 6 | -30 | 11 | 46 | 5.54 |  |
| **L, Inferior Frontal Gyrus** | 47 | -24 | 23 | -9 | 5.18 | 432 |
| **L, Precentral Gyrus** | 4 | -36 | -18 | 48 | 4.07 | 405 |
| **L, Precentral Gyrus** | 44 | -53 | 7 | 13 | 4.18 | 351 |
| **L, Paracentral Lobule** | 31 | -3 | -30 | 43 | 6.98 | 71631 |
| **L, Precuneus** | 7 | -6 | -71 | 39 | 6.87 |  |
| **L, Inferior Parietal Lobule** | 40 | -50 | -33 | 43 | 6.69 |  |
| **L, Superior Temporal Gyrus** | 22 | -50 | 14 | -3 | 4.61 |  |
| **L, Superior Temporal Gyrus** | 22 | -59 | -40 | 8 | 5.61 | 2808 |
| **L, Middle Temporal Gyrus** | 21 | -50 | -46 | 8 | 5.14 |  |
| **L, Middle Temporal Gyrus** | 37 | -53 | -50 | -3 | 4.82 |  |
| **L, Middle Temporal Gyrus** | 39 | -45 | -69 | 17 | 5.31 | 891 |
| **L, Middle Temporal Gyrus** | 39 | -45 | -74 | 26 | 4.25 |  |
| **L, Fusiform Gyrus** | 37 | -33 | -47 | -15 | 4.17 | 432 |
| **L, Culmen** | 30 | -12 | -41 | -6 | 5.26 | 729 |
| **L, Posterior Lobe** | 37 | -33 | -56 | -15 | 4.04 |  |
| **L, Cingulate Gyrus** | 32 | -3 | 22 | 40 | 4.75 |  |
| **L, Anterior Cingulate** | 24 | 0 | 16 | 24 | 6.81 | 4536 |
| **L, Insula** | 13 | -42 | 12 | 2 | 4.74 | 756 |
| **L, Caudate** |  | -6 | 9 | 5 | 8.15 |  |
| **L, Lateral Globus Pallidus** |  | -12 | 6 | 0 | 7.66 |  |
| **R, Superior Frontal Gyrus** | 9 | 33 | 45 | 28 | 4.28 |  |
| **R, Middle Frontal Gyrus** | 10 | 33 | 48 | 20 | 4.50 | 972 |
| **R, Postcentral Gyrus** | 20 | 48 | -24 | 45 | 4.31 |  |
| **R, Parietal Lobe** | 7 | 27 | -47 | 55 | 5.37 | 1107 |
| **R, Inferior Parietal Lobule** | 40 | 33 | -38 | 54 | 4.47 |  |
| **R, Superior Temporal Gyrus** | 22 | 53 | 12 | 2 | 5.55 | 729 |
| **R, Fusiform Gyrus** | 37 | 45 | -56 | -15 | 4.17 |  |
| **R, Culmen** | 37 | 27 | -53 | -15 | 5.16 | 999 |
| **R, Cingulate Gyrus** | 32 | 3 | 25 | 26 | 5.66 |  |
| **R, Caudate** |  | 9 | 6 | 5 | 8.42 | 18900 |

Note: One-sample t-test at *p* *<* 0.05, FWE cluster-corrected.
