# Supplementary material for: Bilinguals Use Language-Control Brain Areas More Than Monolinguals to Perform Non-Linguistic Switching Tasks
Source: PLoS One. 2013 Sep 13;8(9):e73028. doi: 10.1371/journal.pone.0073028 (PMC3772880; doi:10.1371/journal.pone.0073028)
Supplement: Table S2 — Region of interest showing increased activation for bilinguals compared with monolinguals. (DOCX) [file pone.0073028.s004.docx]

| **BILINGUALS > MONOLINGUALS** | | | | | | |
| --- | --- | --- | --- | --- | --- | --- |
| **Activated regions** | **Brodmann**  **areas** | **Talairach coordinates** | | | **T-value** | **Cluster size (mm³)** |
|  |  | **x** | **y** | **z** |  |  |
| **L, Medial Frontal Gyrus** | 10 | -15 | 47 | 11 | 3.53 | 486 |
|  |  | -21 | 41 | 3 | 3.39 |  |
| **L, Inferior Frontal Gyrus** | 44 | -53 | 10 | 19 | 3.35 | 432 |
| **L, Caudate** |  | -21 | -28 | 29 | 4.03 | 351 |
| **L, Caudate** |  | -9 | 9 | 11 | 3.64 | 432 |
| **R, Middle Frontal Gyrus** | 8 | 21 | 25 | 37 | 4.02 | 567 |
| **MONOLINGUALS > BILINGUALS** | | | | | | |
| **R, Supramarginal Gyrus** | 40 | 62 | -42 | 33 | 3.87 | 270 |

Note: Two-sample t-test at *p* < 0.005, uncorrected.
